# Influence of parameter perturbations on the reachability of therapeutic target in systems with switchings

**DOI:** 10.1186/s12938-017-0360-9

**Published:** 2017-08-18

**Authors:** Magdalena Ochab, Krzysztof Puszynski, Andrzej Swierniak

**Affiliations:** Silesian University of Technology, Akademicka 16, Gliwice, Poland

**Keywords:** Biological model, Switches, Piece-wise continuous nonlinear models

## Abstract

**Background:**

Examination of physiological processes and the influences of the drugs on them can be efficiently supported by mathematical modeling. One of the biggest problems is related to the exact fitting of the parameters of a model. Conditions inside the organism change dynamically, so the rates of processes are very difficult to estimate. Perturbations in the model parameters influence the steady state so a desired therapeutic goal may not be reached. Here we investigate the effect of parameter deviation on the steady state in three simple models of the influence of a therapeutic drug on its target protein. Two types of changes in the model parameters are taken into account: small perturbations in the system parameter values, and changes in the switching time of a specific parameter. Additionally, we examine the systems response in case of a drug concentration decreasing with time.

**Results:**

The models which we analyze are simplified, because we want to avoid influences of complex dynamics on the results. A system with a negative feedback loop is the most robust and the most rapid, so it requires the largest drug dose but the effects are observed very quickly. On the other hand a system with positive feedback is very sensitive to changes, so small drug doses are sufficient to reach a therapeutic target. In systems without feedback or with positive feedback, perturbations in the model parameters have a bigger influence on the reachability of the therapeutic target than in systems with negative feedback. Drug degradation or inactivation in biological systems enforces multiple drug applications to maintain the level of a drug’s target under the desired threshold. The frequency of drug application should be fitted to the system dynamics, because the response velocity is tightly related to the therapeutic effectiveness and the time for achieving the goal.

**Conclusions:**

Systems with different types of regulation vary in their dynamics and characteristic features. Depending on the feedback loop, different types of therapy may be the most appropriate, and deviations in the model parameters have different influences on the reachability of the therapeutic target.

## Background

### Biological switches

A significant part of biological processes proceed in a switch-like manner. A typical example of such step changes in biological systems is related to application of a therapeutic drug; after drug administration, especially through injection, specific processes are activated and intracorporeal conditions change in a sudden, step-like manner. When a drug is administered to a patient (orally or by a drip-bag), the number of drug molecules in the body increases rapidly and drug-induced processes start suddenly. The level of active drug cannot be maintained at a steady level due to processes such as transport of drug molecules through the organism, import and export by cells, binding to other molecules, and drug degradation or inactivation [1]. Administration of a drug is intended to enforce specific processes such as inactivation or degradation of a protein target or blockade of inter- or extra-cellular transport [2]. Consequently, the activity of a drug should be included in a model in a multiplicative manner: drug molecules influence the rate of the processes related to the number of protein or other molecular targets. Instead of considering the drug application as an input to the model, we propose considering it as an abrupt change of the parameter values; in this case the drug accelerates the process of protein degradation. Other examples of biological processes which proceed in a switch-like manner are gene activation and deactivation [3] and transmembrane transport through (divided by) ion channels [4]. These and many other biological processes can be effectively modeled as systems with switches in the parameter values.

### Parameter changes

The intra- and inter-cellular environments are very complex, containing many different types of molecules both non-organic and organic such as carbohydrates, proteins, lipids, and nucleic acids. Because of intra- and extra-cellular processes such as export and import, production and degradation of proteins and their modification by phosphorylation, ubiquitylation, or glycosylation, the chemical and physical conditions inside the cell change dynamically [3]. Similarly, cell growth during the cell cycle influences the concentrations of all the substances inside the cell and thereby the rate of all processes. Consequently, the rates of cellular processes are dynamically changing [5]. The building of mathematical models requires exact determination of model parameters. The parameters for biological processes are calculated or estimated using the results of biological experiments and thus depend highly on the biological, chemical and physical conditions in cells. Sensitivity analysis of models is usually employed to test their vulnerability to small perturbations of parameters [6], but when a change of the parameter values is large the model outputs may be significantly different. In systems with switching, the problem of parameter perturbation can cause even greater inaccuracy due to cumulation of two effects: first, the initial state before the switch can be significantly altered, and second, the parameters in the model after a switch are biased. Consequently, the dynamics of such models can be significantly different from the real dynamics. This factor plays an essential role, especially in situations when therapy with minimized doses of a drug is considered.

#### Drug degradation

In our preliminary study we neglect the pharmacodynamics and pharmacokinetics and assume that the concentration of a drug in the organism is maintained at a stable level. This approach is useful when examining the internal properties of the system, although in real-life systems the problem of estimating drug level and its influence on the organism play an essential role. The problem of drug pharmacodynamics and pharmacokinetics has been widely examined and discussed (see e.g. [7–9]). Mechanism-based Pharmacokinetic/Pharmacodynamic (PK-PD) models contain expressions to quantitatively characterize processes such as drug distribution at the target site and drug binding, activation, and inactivation or excretion. Moreover, there are many other factors which can influence the therapeutic or toxic activity of a drug such as the problem of drug-drug interactions which affect the pharmacokinetics , by influencing gastro-intestinal absorption, occupying binding sites on a protein, or competing for drug-metabolizing enzyme systems are described exhaustively by Kristensen [10]. Nevertheless, an interesting problem of pharmacokinetics arises in a system with multiple drug dosage. Clinical research on drug administration supported by mathematical modeling is useful in determining the efficacy and safety of proposed therapies [11]. Model parameters should be fitted for a specific drug, as in our previous work where based on the results published by Zhang et al. [12] we created model equations describing the active and total concentrations for the experimental antitumour drug Nutlin [13]. In the present work we do not consider any specific drug, so we use the previously published equations and slightly modify the parameters to obtain desirable dynamics. The kinetics of the protein degradation is not described by the Michaelis–Menten kinetics, because the number of active molecules of a drug is comparable to the number of protein target molecules, so the process of increased degradation of a target protein by a drug can be described by the mass-action law.

#### Regulation of protein production

Modeling of biological systems demands a special approach due to their very high complexity. The great majority of these systems are modeled by deterministic algorithms, although in some cases a stochastic approach is more suitable especially when a low number of reacting molecules is involved such as in gene switching processes. Parameter fluctuations may be also considered as stochastic changes of parameter values [14] or modeled as a bounded stochastic process [15–17]. However, here we use a deterministic approach despite the existence of gene states in our models and we postpone investigation of stochastic models, since the majority of published models are still deterministic.

The use of a switched system for modeling biological processes is not a new approach, although to our knowledge the achievability of a therapeutic target in systems with parameter perturbation has not been considered. There have been attempts to use piecewise linear systems to model highly non-linear processes, in which the dependencies between molecules are described by the switching rules which enforce changes in the model structure or parameter values [18–20]. Similar methods were presented by de Jong and co-workers, who proposed qualitative simulation of regulatory networks using piecewise linear models [21, 22]. A different application of switched system was presented by Parise et al. [23], where a stochastic model of a controlled gene expression system is discussed focussing on the achievability region for the protein produced from the gene by solving an optimization problem.

## Methods

### Proposed models

In this work we investigate the problem of the influence of parameter perturbations on a medical therapy designed to decrease the number of active molecules of a target protein. In the first part of this paper three models are considered which all consist of three variables: *G*—the gene state, *R*—the number of mRNA molecules transcribed from the gene, and *A*—the number of molecules of the protein *A*. In the second part the model is expanded by two more equations, which describe the total and active drug levels. The model without a feedback loop is a well-known model of protein production, and the model with a negative feedback loop was published in [24]. Additionally, we create a model with a positive feedback loop to compare the influence of different types of autoregulation. The parameter values are taken partially from our previous work [24, 25], where we presented some preliminary results. Here the positive feedback loop is created in a different way, and we further examine the case when a drug is degraded or inactivated. The number of target protein molecules in the steady state before drug application is established arbitrarily at 160,000 molecules and the therapeutic goal is to reduce this level to below 70,000 molecules.

The basic system without any feedback loop is described by the following equations:

1 $$\begin{aligned} \frac{dG}{dt} = q_{a} * (N_A - G(t)) - q_{d1}* G(t), \end{aligned}$$

2 $$\begin{aligned} \frac{dR}{dt} = t_1 * G(t) - d_1 * R(t), \end{aligned}$$

3 $$\begin{aligned} \frac{dA}{dt} = t_2*R(t) - d_2 * A(t) . \end{aligned}$$

To decrease the protein level below the required threshold, we apply a drug which induces its degradation or inactivation. In the first part of our approach we neglect pharmacokinetics and pharmacodynamics, so the drug concentration is maintained at a stable level. The drug application is considered as the parameter switch because it induces a step change in the value of the rate of degradation of protein *A*. The basic therapeutic goal is to maintain the level of protein *A* under the required threshold. After the switching time ts Eq. (3) changes to the following form:

4 $$\begin{aligned} \frac{d A}{dt} = t_2*R(t) - (d_2+d_3*DRUG)*A(t). \end{aligned}$$

**Fig. 1:**
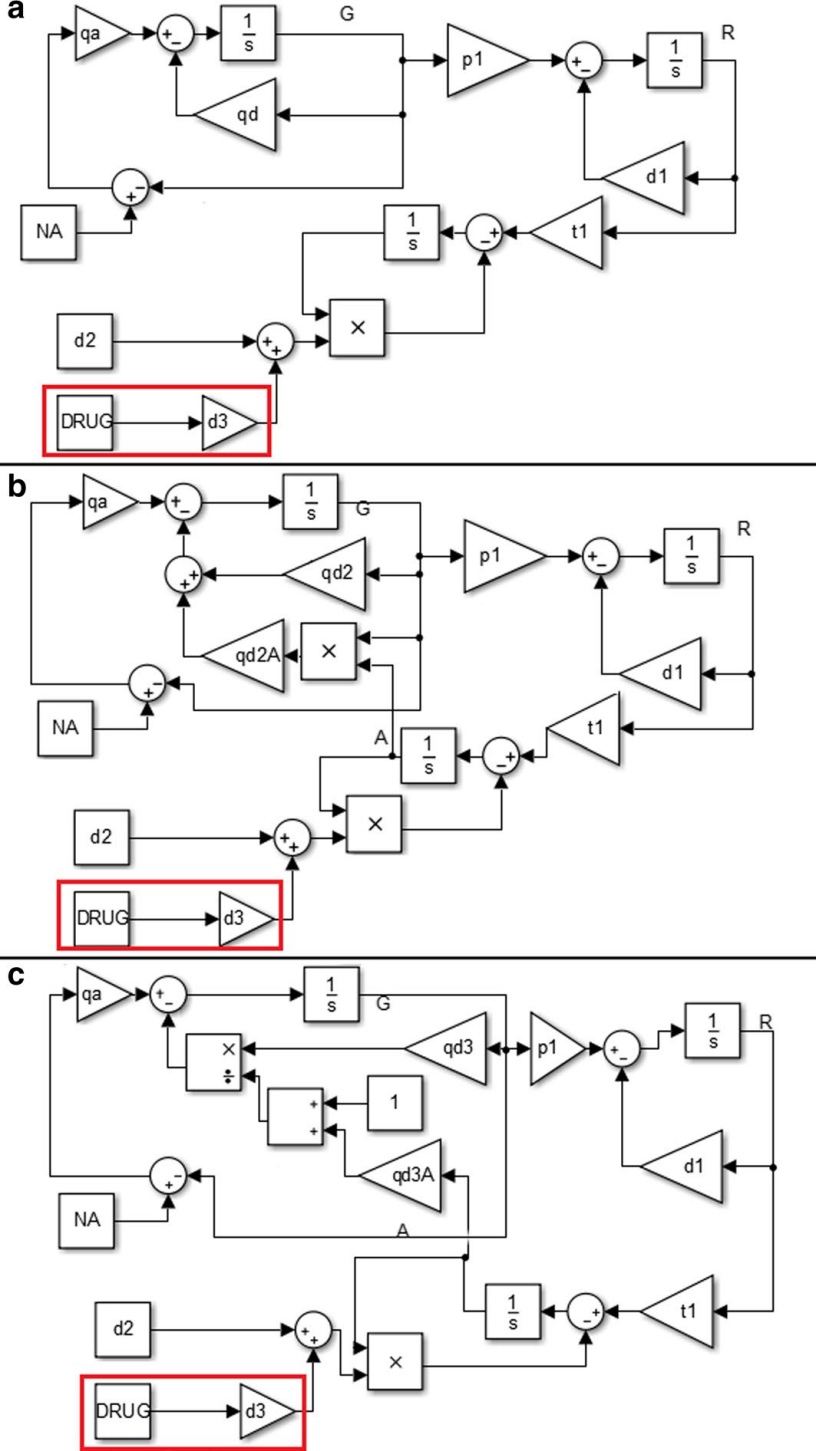
Schematic diagrams of the models. **a** Model without feedback loops. **b** Model with negative feedback loop in which protein *A* represses its own gene. **c** Model with positive feedback loop in which protein *A* blocks repression of its own gene. The *red box* contains the step added after switching and related to the drug application.

The structure of this model is presented in Fig.  1a.

Biological processes are usually regulated by a variety of feedback loops, which are mainly negative although positive loops are also well known. We propose two additional models, the first with a negative feedback loop and the second with a positive feedback loop. In the first model protein *A* acts as a repressor of its own transcription causing it to increase the deactivation rate of its gene. The equation which describes the gene state has the following form:

5 $$\begin{aligned} \frac{dG}{dt} = q_{a} * (N_A - G(t)) - q_{d2} *G(t) - q_{d2A} * G(t) *A(t). \end{aligned}$$

The structure of the model with a negative feedback loop is presented in Fig. 1b.

The model with a positive feedback loop is created by double negation where protein *A* blocks repression of the gene responsible for its production. As a result transcription of the gene increases and subsequently the production of mRNA and protein is higher. The first equation of this model describes the gene state and its dependency on the number of protein molecules:

6 $$\begin{aligned} \frac{dG}{dt} = q_{a} * (N_A - G_A(t))- \frac{q_{d3}}{1+q_{d3A} * A} * G(t). \end{aligned}$$

The structure of the model with a positive feedback loop is presented in Fig. 1c. To maintain a similar protein level of $\sim$160,000 molecules in all the models we fitted the spontaneous gene deactivation rates (these are noted correspondingly by $q_{d1}$, $q_{d2}$ and $q_{d3}$). Other parameters are the same in all the models (see Table 1).

**Table 1:** Values of the model parameters

Parameter	Description	Value	Unit
$q_{a}$	Gene activation	$2.78*10^{-4}$	1/s
$q_{d1}$	Gene deactivation	$2.78*10^{-4}$	1/s
$q_{d2}$	Spontaneous gene deactivation (negative feedback)	$2.78*10^{-5}$	1/s
$q_{d2A}$	Protein induced gene deactivation (negative feedback)	$1.565*10^{-9}$	1/s
$q_{d3}$	Spontaneous gene deactivation (positive feedback)	$2.4*10^{-3}$	1/s
$q_{d3A}$	Protein induced gene activation (positive feedback)	$4.8*10^{-5}$	1/s
$t_1$	mRNA transcription	0.05	Molecules/s
$t_2$	Protein translation	0.1	1/s
$d_1$	mRNA degradation	$1.5*10^{-4}$	1/s
$d_2$	Protein degradation	$2.0822*10^{-4}$	1/s
$d_3$	Protein degradation dependent on drug	$3*10^{-9}$	1/s
*DRUG*	Number of drug molecules	$1.4*10^3$	Molecules
$N_A$	Number of alleles	2	Alleles

### Steady states

The steady state equations for all variables in the model without a feedback loop are as follows:

$$\begin{aligned} {G_{ss}}&= \frac{N_A * q_{a}}{q_{a} + q_{d1}}, \\ R_{ss}&= \frac{t_1 * N_A * q_{a}}{d_1*(q_{a} + q_{d1})}, \\ A_{ss}&= \frac{t_2*t_1 * N_A * q_{a}}{d_2*d_1*(q_{a} + q_{d1})}. \end{aligned}$$

For the models with a positive or negative feedback loop the formulas for the steady state are much more complicated. Derivation includes solution of the square equations, but because one root is positive and the other is always negative there is only one steady state. The steady state for the model with a negative feedback loop is described by the equations:

$$\begin{aligned} {G_{ss}}&= \frac{-(q_a+q_{d2}) + \sqrt{(q_a+q_{d2})^2 + 4*N_A*q_a*q_{d2A}*\frac{t_2*t_1}{d_1*d_2}}}{2*\frac{t_2*t_1}{d_1*d_2}*q_{d2A}},\\ R_{ss}&= \frac{t_1 }{d_1} * \frac{-(q_a+q_{d2}) + \sqrt{(q_a+q_{d2})^2 + 4*N_A*q_a*q_{d2A}*\frac{t_2*t_1}{d_1*d_2}}}{2*\frac{t_2*t_1}{d_1*d_2}*q_{d2A}}, \\ A_{ss}&= \frac{t_2*t_1}{d_2*d_1} * \frac{-(q_a+q_{d2}) + \sqrt{(q_a+q_{d2})^2 + 4*N_A*q_a*q_{d2A}*\frac{t_2*t_1}{d_1*d_2}}}{2*\frac{t_2*t_1}{d_1*d_2}*q_{d2A}}. \end{aligned}$$

For the model with a positive feedback loop the formulas for the steady state are as follows:

$$\begin{aligned} G_{ss} &= {} \frac{-(q_a*N_A*q_{d3A}*\frac{t_1*t_2}{d_1*d_2}-q_a-q_{d3})}{-2*q_a*q_{d3A}*\frac{t_1*t_2}{d_1*d_2}} \\ & \quad - \frac{ \sqrt{(q_a*N_A*q_{d3A}*\frac{t_1*t_2}{d_1*d_2}-q_a-q_{d3})^2 + 4 * q_a^2 * q_{d3A}*N_A *\frac{t_1*t_2}{d_1*d_2}}}{-2*q_a*q_{d3A}*\frac{t_1*t_2}{d_1*d_2}} \\ R_{ss}&= {} \frac{-(q_a*N_A*q_{d3A}*\frac{t_1*t_2}{d_1*d_2}-q_a-q_{d3})}{-2*q_a*q_{d3A}*\frac{t_1*t_2}{d_1*d_2}} \\ & \quad - \frac{ \sqrt{(q_a*N_A*q_{d3A}*\frac{t_1*t_2}{d_1*d_2}-q_a-q_{d3})^2 + 4 * q_a^2 * q_{d3A}*N_A *\frac{t_1*t_2}{d_1*d_2}}}{-2*q_a*q_{d3A}*\frac{t_2}{d_2}} \\ A_{ss} &= {} \frac{-(q_a*N_A*q_{d3A}*\frac{t_1*t_2}{d_1*d_2}-q_a-q_{d3})}{-2*q_a*q_{d3A}*\frac{t_1*t_2}{d_1*d_2}} \\ & \quad - \frac{ \sqrt{(q_a*N_A*q_{d3A}*\frac{t_1*t_2}{d_1*d_2}-q_a-q_{d3})^2 + 4 * q_a^2 * q_{d3A}*N_A *\frac{t_1*t_2}{d_1*d_2}}}{-2*q_a*q_{d3A}} \end{aligned}$$

For the positive feedback loop there exists only one steady state, because the second solution of the quadratic equation is always negative. To calculate the steady states after drug application the parameter $d_2$ should be replaced by the formula ($d_2 + d_3 *DRUG$). Drug application change the values of the variables in the steady states but does not affect the stability of the models. The calculated values of the steady states in each case are presented in Table 2. In each case we apply the same dose of drug equal to 140,000 molecules.

**Table 2:** Values of the steady states in models with or without drug application (drug dose 140,000 molecules)

Model	Drug	*G*	*R*	*A*
		Gene	mRNA	Protein
Without feedback	−	1.0000	333.33	160,087
Without feedback	+	1.0000	333.33	53,060
Negative feedback	−	0.9996	333.19	160,020
Negative feedback	+	1.3346	444.85	70,811
Positive feedback	−	1.0053	335.09	160,930
Positive feedback	+	0.3656	121.87	19,399

### Simulations

All the simulations were performed by a deterministic algorithm, specifically a Runge–Kutta 4th order algorithm. The results obtained cannot be treated as time courses of the number of molecules in one cell, because we do not take into account the stochastic nature of the processes. However, the deterministic approach enables prediction of the mean behavior of cells, which can be considered as the response of a large cell population like a whole organism. The simple control objective is to keep the protein level $A(t)<Th$, where *Th* is the required threshold. To ensure effective therapy, the protein level must be maintained under the threshold until the end of the simulation. The time when this target is achieved (when *A* goes below *Th*) is not specified, but a faster therapeutic effect is preferable.

We consider the influence of two different types of perturbations: the uncertainty of the parameter values, and the inaccuracy in estimation of switching time. In the case of deviation of the parameter values we focus on the parameter $q_a$, which represents the gene’s activation. This rate is changing dynamically, because the concentration of transcription factors fluctuates continuously. This parameter is very difficult to estimate and its value can change during a cell’s life. Further, its value varies from person to person and depends on the physiological conditions at the specific moment. As shown in [24], gene switching time may be crucial for the success or failure of therapy. Inaccuracy in the switching time is the result of delays in drug application and differences in the rate time of the drug’s distribution in the organism.

**Table 3:** The values of the parameters for model with drug degradation

Parameter	Description	Value	Unit
$p_{drug}$	Dose conversion factor	$7.5*10^{-9}$	M/(mg/kg)
*U*	Drug dose rate	5	mg/(kg s)
$K_a$	Equilibrium association rate	$8.5*10^{4}$	1/M
$B_{max}$	Concentration of protein binding sites	$1.1*10^{-3}$	M
$\delta$	Drug elimination rate	$5.4*10^{-4}$	1/s
*i*	Drug import to cell	$1.27*10^{8}$	molec/(s M)
*e*	Drug export from cell	$5*10^{-3}$	1/s

## Results

### Results for models without drug degradation

**Fig. 2:**
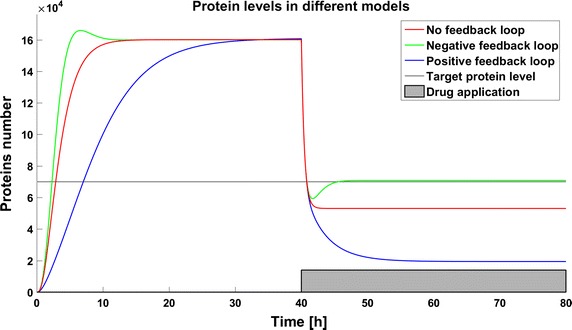
Time courses of protein levels in different models. Number of target protein molecules after drug application in the model without feedback loop (*red line*), the model with negative feedback loop (*green line*), and the model with positive feedback loop (*blue line*)

First we examine the response of models after application of the same dose of a drug. We postulate that the therapeutic target is reached if the level of the target protein is less than 70,000 molecules. This target is reached for a drug dose of 140,000 molecules in both the system without any feedback loops and in that with positive feedback, but the same drug dose results in a much lower level of the target protein in the system with a positive feedback loop. The system with a negative feedback loop is less sensitive to the drug, which reduces the target protein level under the threshold but then stabilizes it above the threshold so that the therapeutic goal is not reached (Fig. 2).

### Parameter changes

**Fig. 3:**
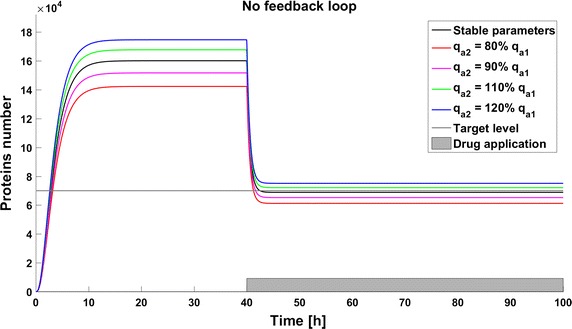
Achievability of the target protein level with changes of the gene activation rate (parameter $q_a$) in the model without feedback loops

**Fig. 4:**
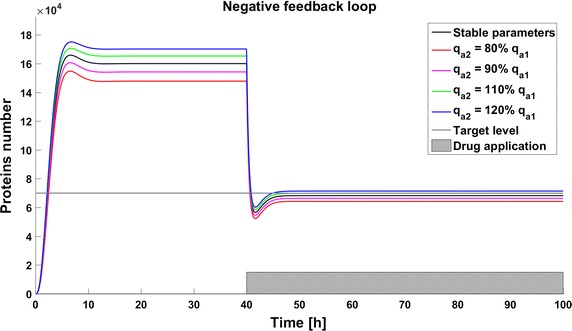
Achievability of the target protein level with changes of the gene activation rate (parameter $q_a$) in the model with negative feedback loop

**Fig. 5:**
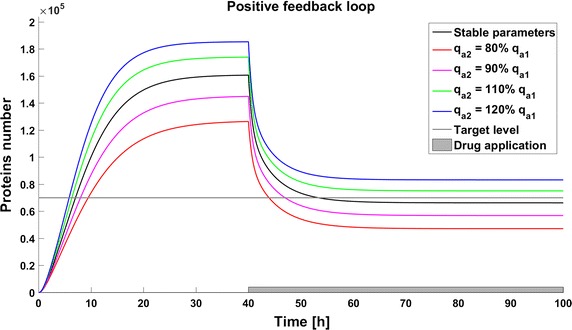
Achievability of the target protein level with changes of the gene activation rate (parameter $q_a$) in the model with positive feedback loop

**Fig. 6:**
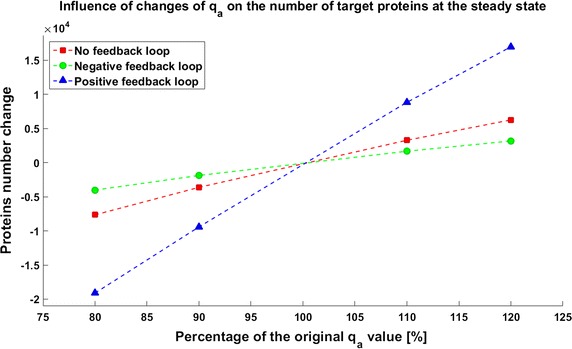
Influences of parameter change on the perturbation of the number of protein molecules in the equilibrium state compared to the nominal in each model

Due to the potential toxicity of drugs we want to minimize their dose, but on the other hand a low drug dose can result in inefficient therapy. Thus determining the optimal drug dose is challenging. We found that for simplified models (without considering complicated drug pharmacokinetic and pharmacodynamics) the effective drug dose is smaller for the model with a positive feedback loop; 40,000 drug molecules are sufficient to decrease the protein level under threshold, whereas 90,000 are needed for the model without any feedback. The model with negative feedback is almost not sensitive to changes in drug administration and the effective dose is very high (150,000 drug molecules). To investigate the influence of parameter perturbations on the achievability of the therapeutic goal, we altered the rate of gene activation $q_a$ to 80, 90, 110, or 120% of the original value. These perturbations have a significant influence on the steady states, and thus in some cases the therapeutic target is not reached. Change of the $q_a$ value results in an altered protein level before and after drug application. In all the systems a reduction of the level of gene activation results in a decreased protein level, so the therapeutic goal is reached. On the contrary, after increasing the value of parameter $q_a$ the protein level increases, so in some cases the therapy with the defined drug dose cannot be efficient (Figs. 3, 4, 5). The steady states of all the models depend on the values of the parameter *qa*, but uncertainty in its value have a different influence for different models. In the model with a negative feedback loop deviations of the gene activation rate have the smallest influence on the steady state (Fig. 6 (green line)) whereas in the model with positive feedback loop the observed perturbations are the greatest (Fig. 6 (blue line)).

### Time of the switch change

**Fig. 7:**
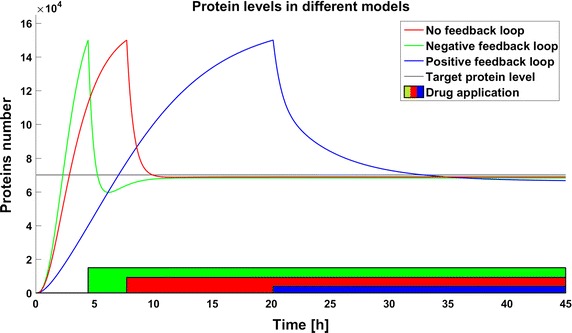
Comparison of the results of models results after drug application on the time of reaching 150,000 protein molecules without perturbation

**Fig. 8:**
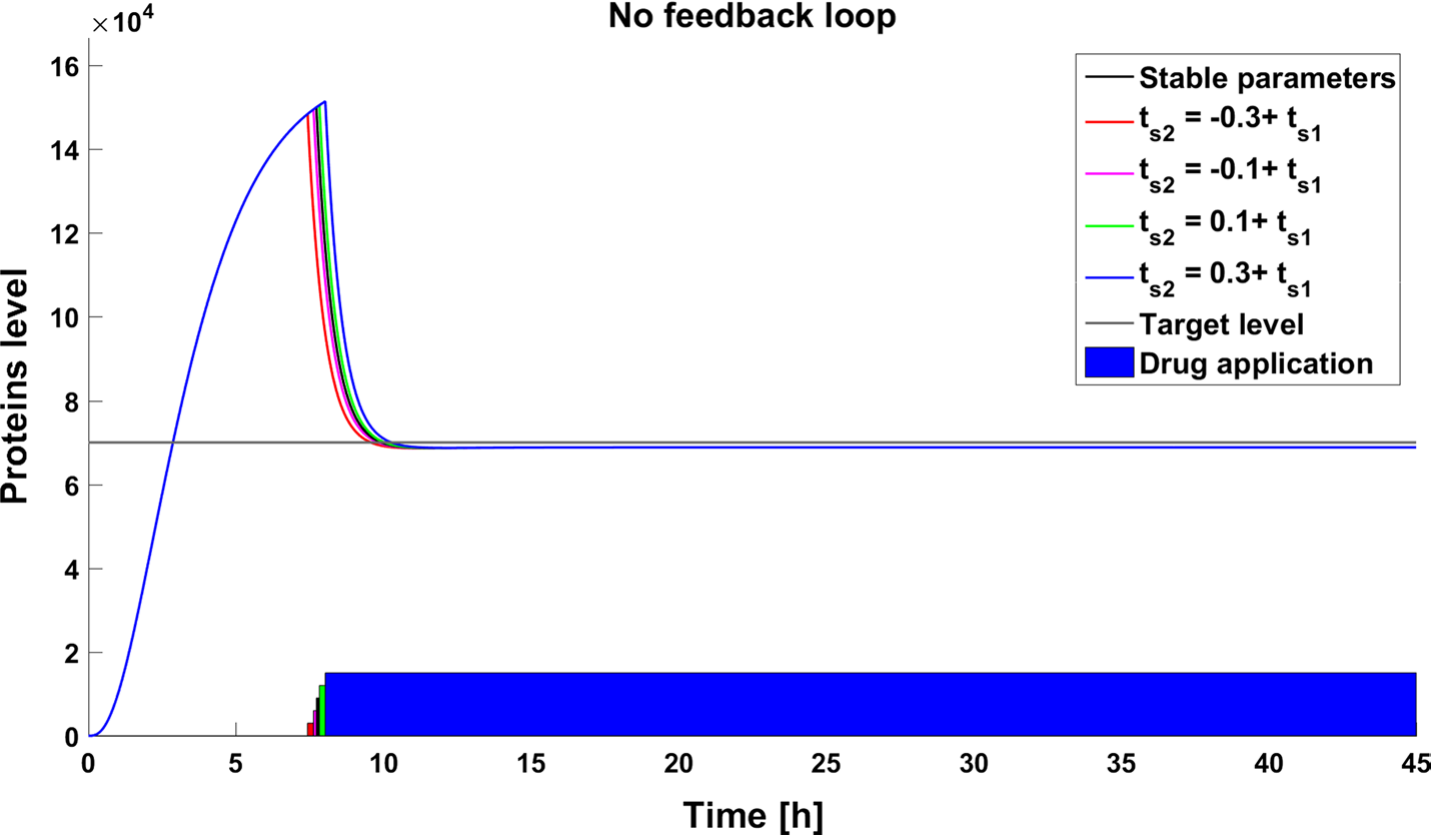
Time to reach the target number of protein molecules with changes of switch time. Model without feedback loops.

**Fig. 9:**
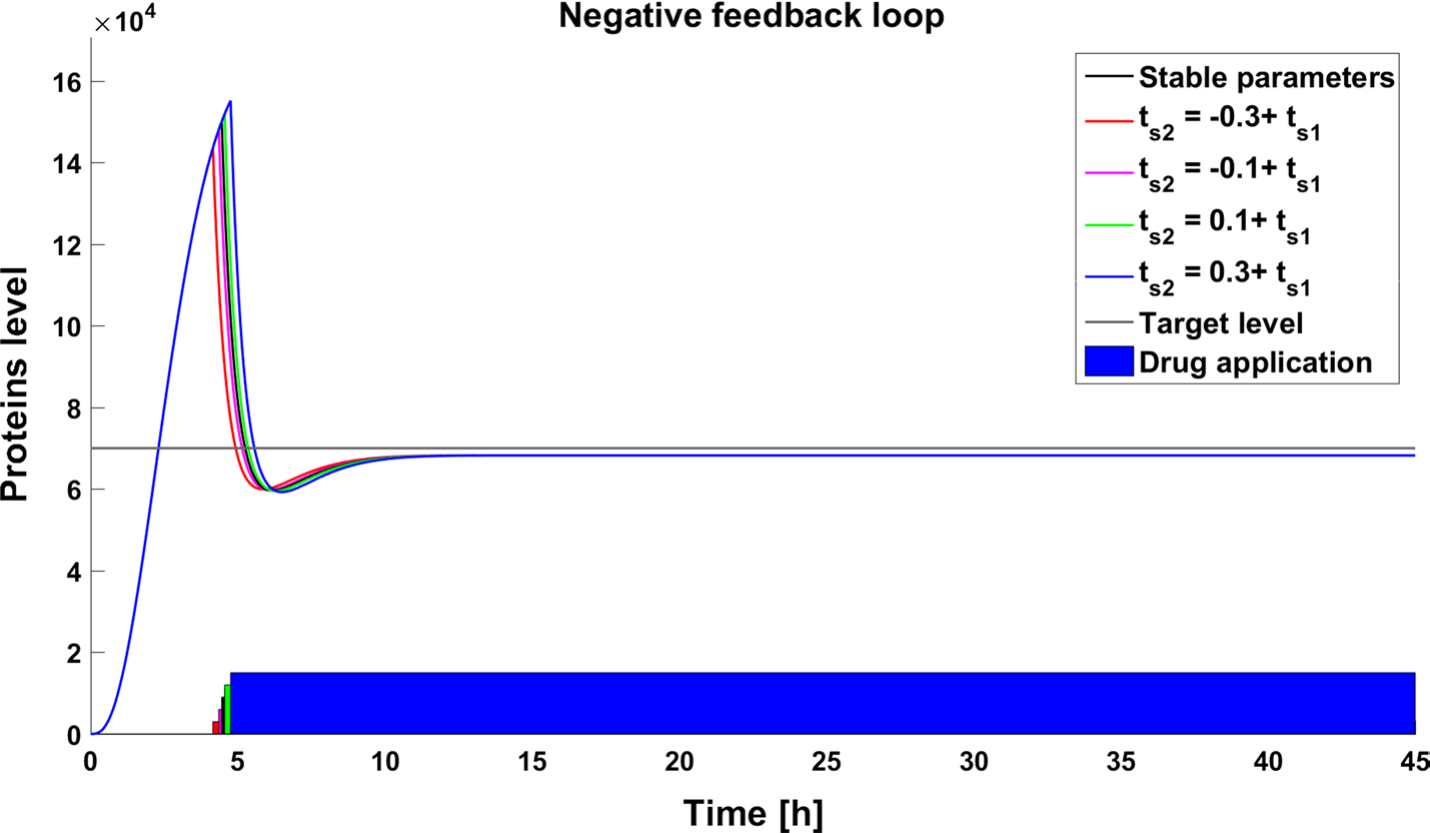
Time to reach the target number of protein molecules with changes of switch time. Model with negative feedback loop

**Fig. 10:**
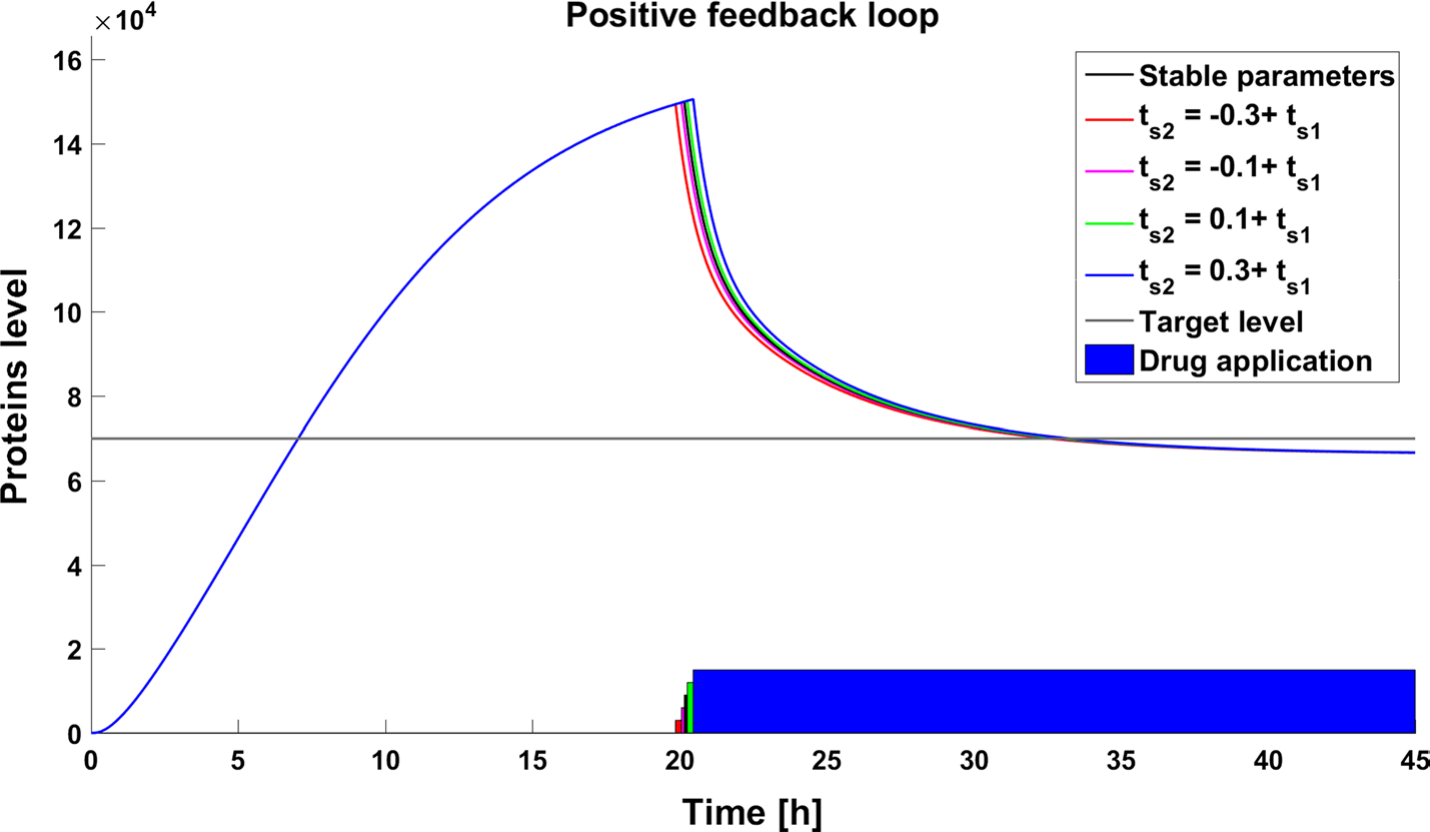
Time to reach the target number of protein molecules with changes of switch time. Model with positive feedback loop

The second type of deviation considered here results from inaccuracy in the switching time. In this experiment the drug application starts when the number of protein molecules reaches 150,000. Consequently, the switching time occurs before stabilization of the the system in the steady state. Such conditions mimic those in real life where a therapy is initiated when some specified biological indicator exceeds a specified level. The differences in dynamics between the created models are presented in Fig. 7. The time to reach the therapeutic target differs significantly for different systems. The dynamics of the model with a negative feedback loop are the fastest, and in this case the response to the drug is much more rapid than in the other cases. The differences in the time to reach the therapeutic goal are the cumulative effect of two factors: the variation in the switching time and the differences in target protein levels at the switching time. The value of the switching time does not affect the target protein numbers in the steady state, but only the time at which the goal is reached. In all cases, the sooner the switch occurs the sooner the goal is reached. In the model without any feedback the steady state is reached 2.3 h after the switching time (Fig. 8). The dynamics in the model with a negative feedback loop are faster than in the other models and the increase and decrease of target protein number is more rapid—the drug application is after 4.5 h of simulation and the therapeutic goal is reached less than 1 h later. However, due to overshoot the steady state is reached after 10 h of simulation (Fig. 9). The slowest changes are observed in the model with a positive feedback loop, where the goal is reached approximately 13 h after drug application (Fig. 10).

### Drug degradation and inactivation

In biological systems like human organisms a stable number of drug molecules is impossible to maintain, because a variety of processes influences the number of active drug molecules. First, application of a drug is a relatively short process (even in the case of intravenous drip it takes no longer than 2 h) and the level of active drug does not increase in a stepwise manner because of the delay in transport to target cells. Moreover, the number of active molecules in target cells depends on their intracellular import and export, and the drug level decreases gradually due to excretion which finally leads to complete removal of drug from the body [26]. We took into account all these factors by adding two more equations to our model, which describe the total amount of drug outside cells and the amount of active drug (not bound to plasma proteins or tissues and thus available for target cells). The equation for the total number of drug molecules consists of a term for the drug application and a term for its removal. The drug application is described by a very simple formula, so it exhibits simplified and very rapid dynamics, while the process of drug removal concerns only molecules not bound to any other particles or tissues, which are denoted by $DRUG_{N}$:

7 $$\begin{aligned} \frac{d DRUG_{tot}(t)}{dt} = p_{drug} * U(t) - \delta * DRUG_{N}(t). \end{aligned}$$

where $DRUG_{tot}(t)$ stands for the total drug number of drug molecules in the body and *U*(*t*) is the drug dose. The number of free drug molecules ($DRUG_{N}(t)$) is defined by the equation:

8 $$\begin{aligned}DRUG_{N} (t) &= \frac{-(1+K_a*B_{max}-K_a*DRUG_{tot}(t))}{2*K_a} \\ & \quad + \frac{\sqrt{(1+K_a*B_{max}-K_a*DRUG_{tot}(t))^2+4*K_a*DRUG_{tot}(t)}}{2*K_a}. \end{aligned}$$

The above equation is derived from the standard equilibrium equation presented in [13]. The parameter $B_{max}$ denotes the concentration of total tissue and plasma protein drug-binding sites and $K_a$ is an equilibrium association constant. The number of active drug molecules depends on the number of free molecules in the organism ($DRUG_N$) and their transport into and out of the cell. We assume that the dynamics of drug transport are very rapid, so we neglect drug degradation inside cells or tissue. The number of drug molecules in cells is described by the equation:

9 $$\begin{aligned} \frac{d DRUG (t)}{dt} = i * DRUG_{N}(t) - e*DRUG(t). \end{aligned}$$

The equations describing the pharmacodynamics and pharmacokinetics were published in our previous paper [13], where we presented the biological justification for the formulas and the parameter values based on the drug Nutlin (Table 3).

**Fig. 11:**
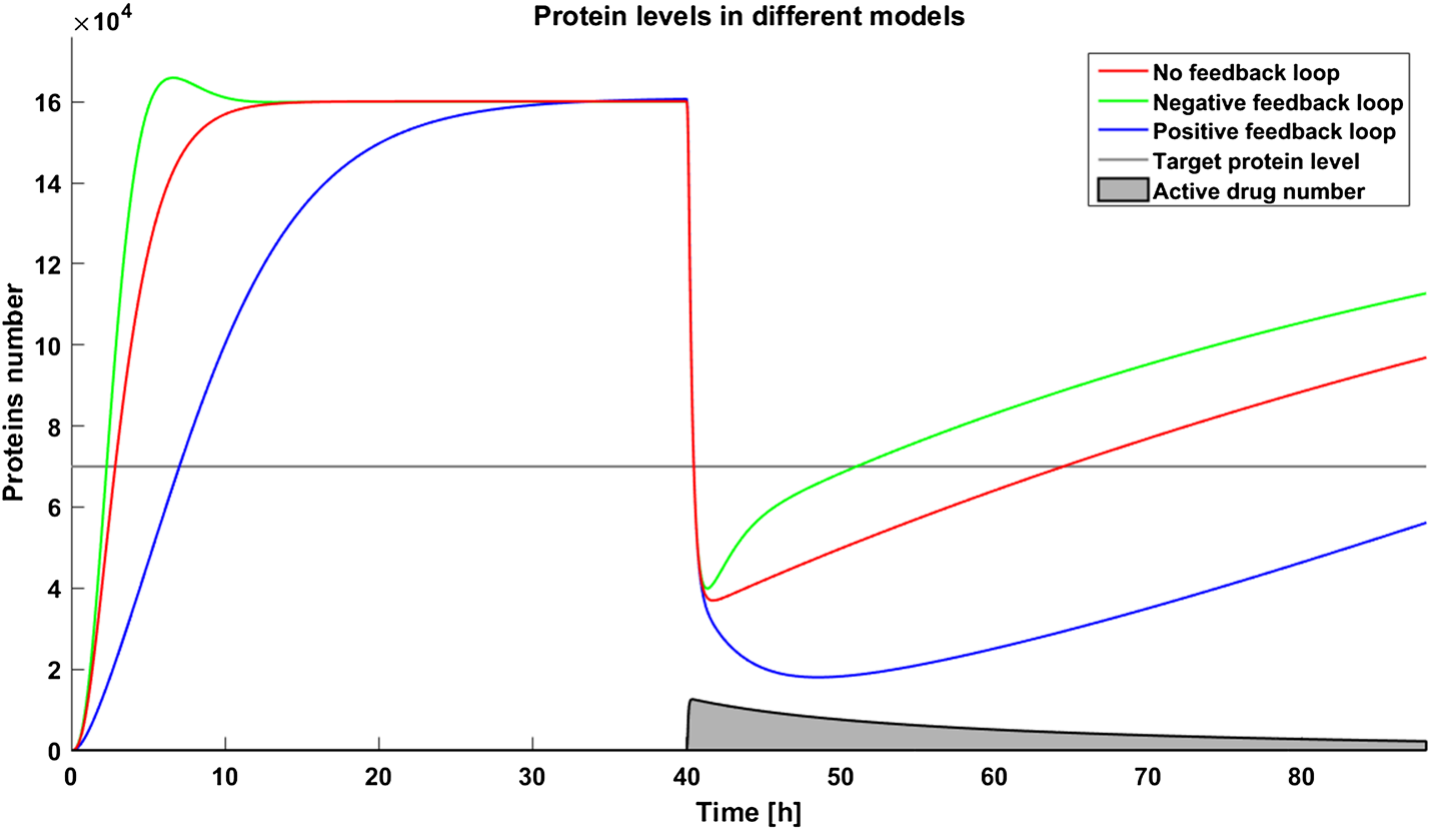
Time courses of the protein levels in different models with drug degradation. Note that in all cases the therapy fails after some time

**Fig. 12:**
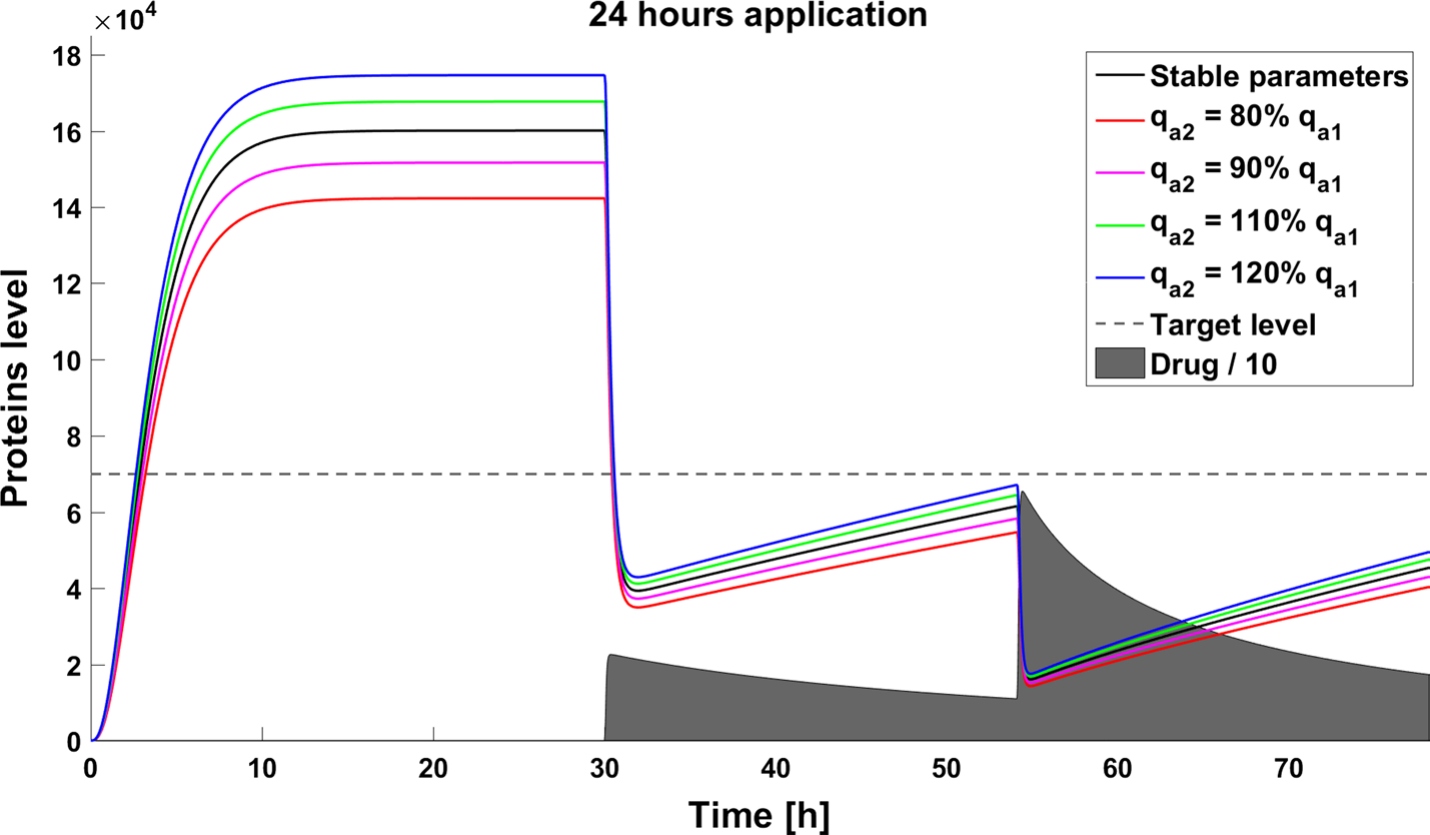
Time course of the level of target protein molecules in the model without a feedback loop with drug degradation after application every 24 h

**Fig. 13:**
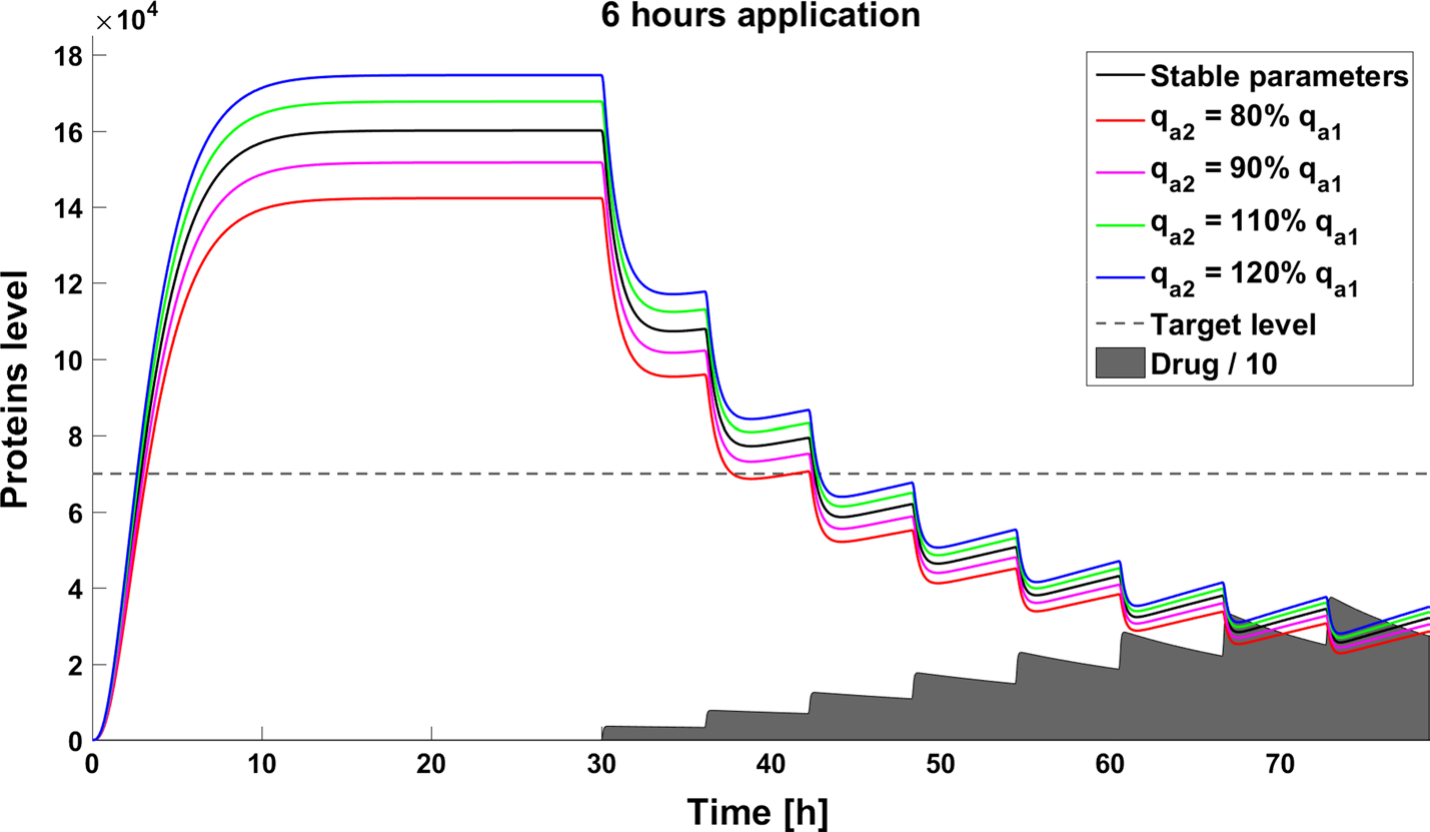
Time course of the level of target protein molecules in the model without a feedback loop with drug degradation after application every 6 h

**Fig. 14:**
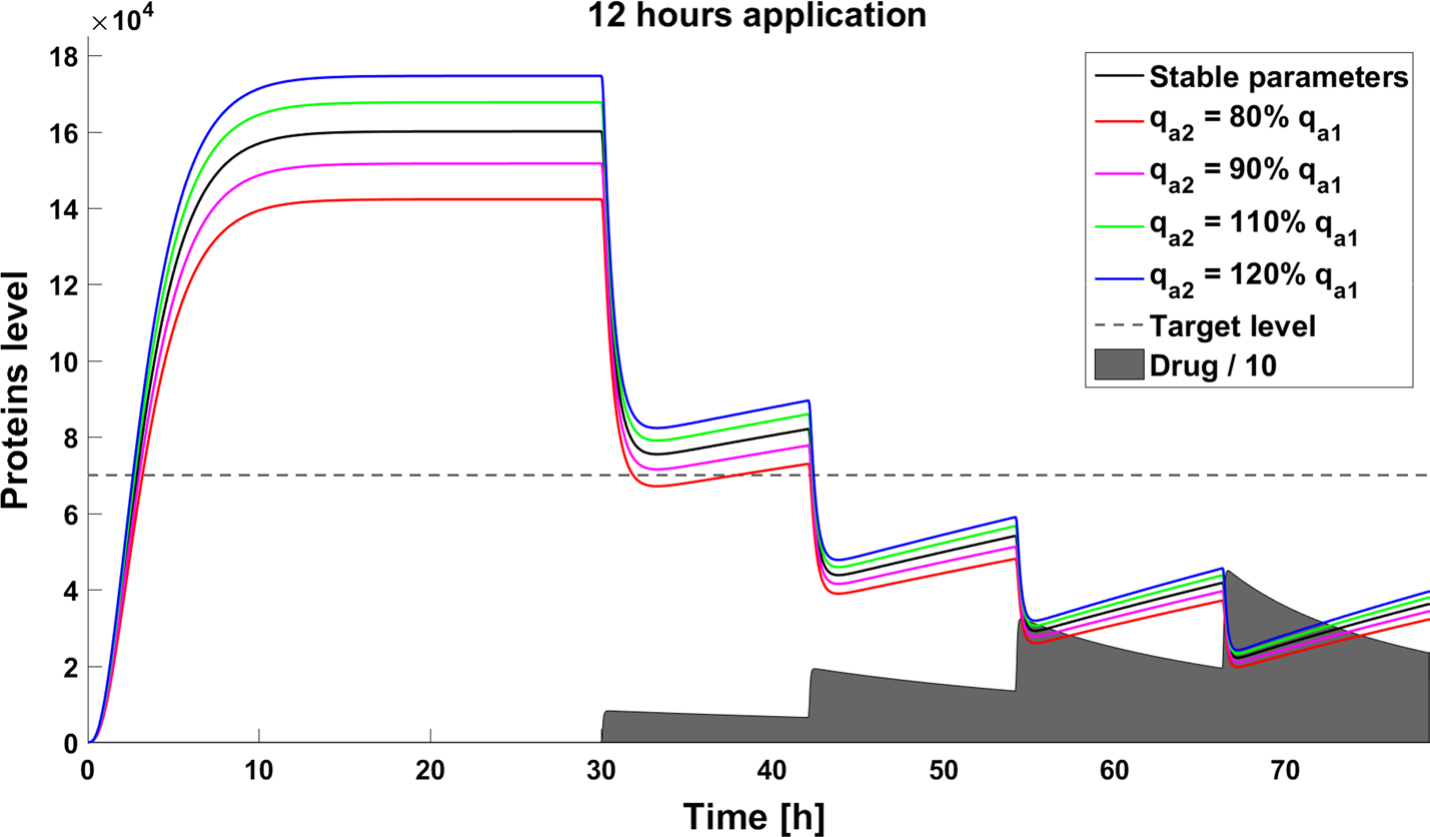
Time course of the level of target protein molecules in the model without a feedback loop with drug degradation after application every 12 h

**Fig. 15:**
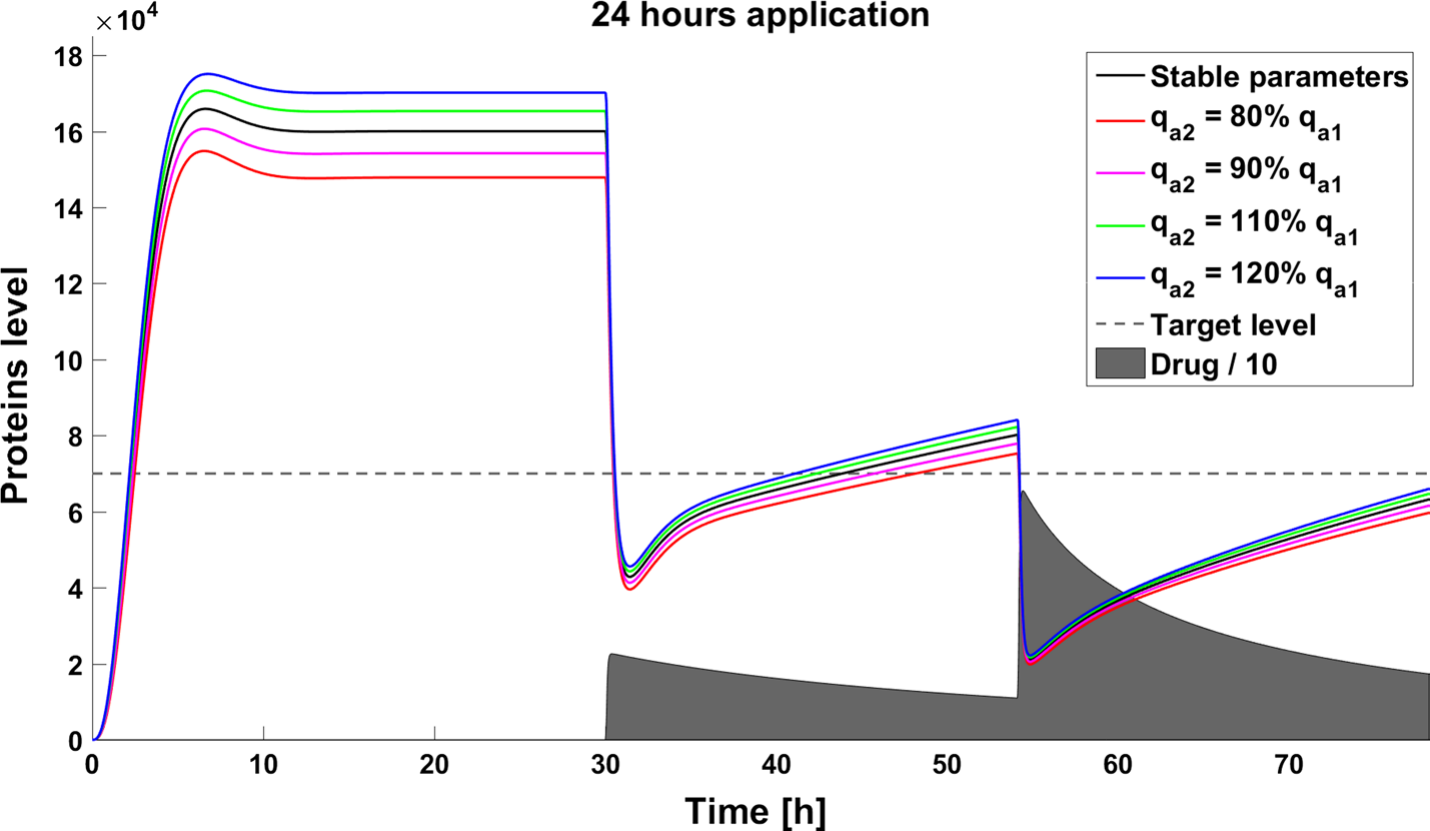
Time course of the level of target protein molecules in the model with a negative feedback loop with drug degradation after application every 24 h

**Fig. 16:**
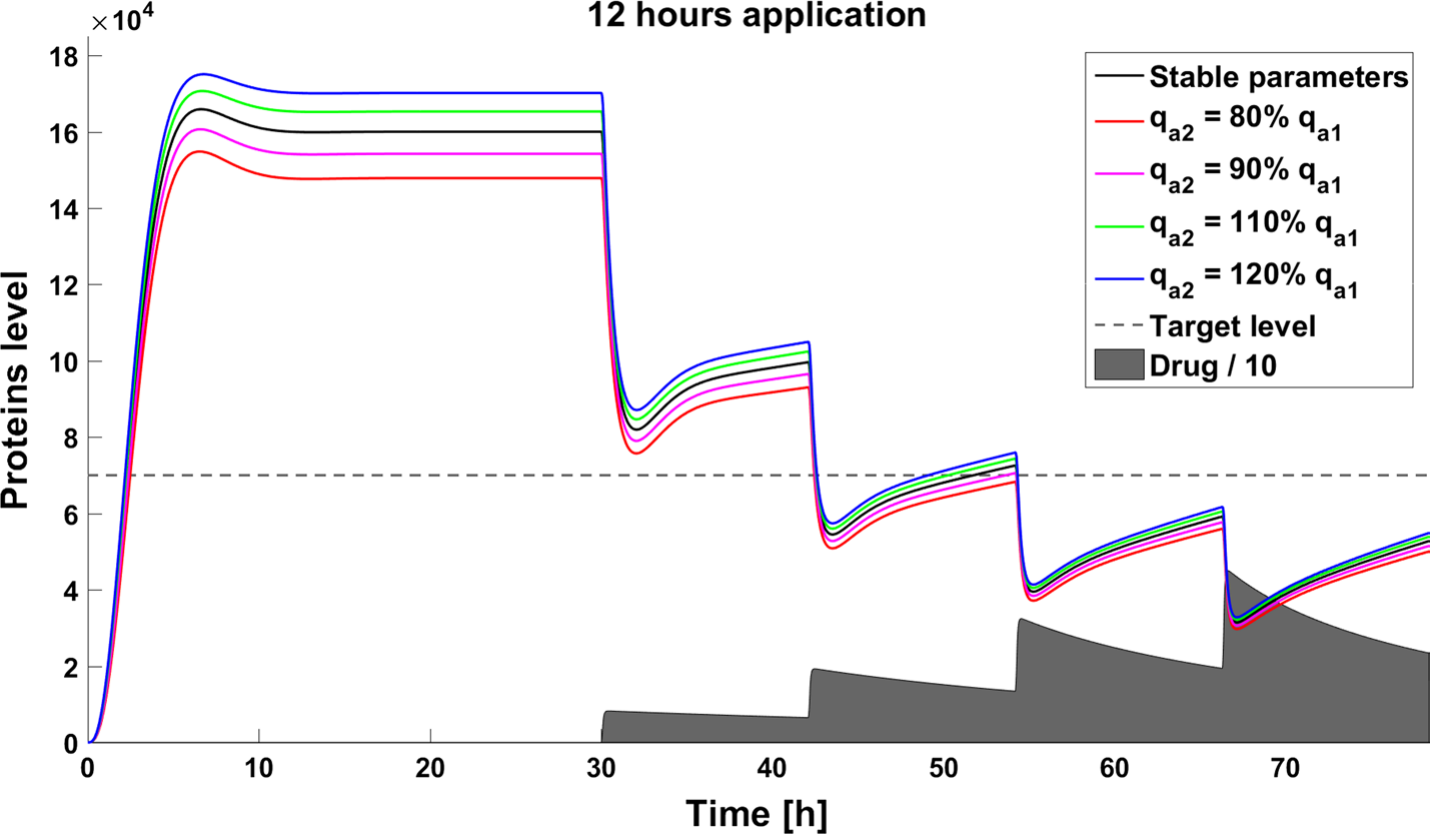
Time course of the level of target protein molecules in the model with a negative feedback loop with drug degradation after application every 12 h

**Fig. 17:**
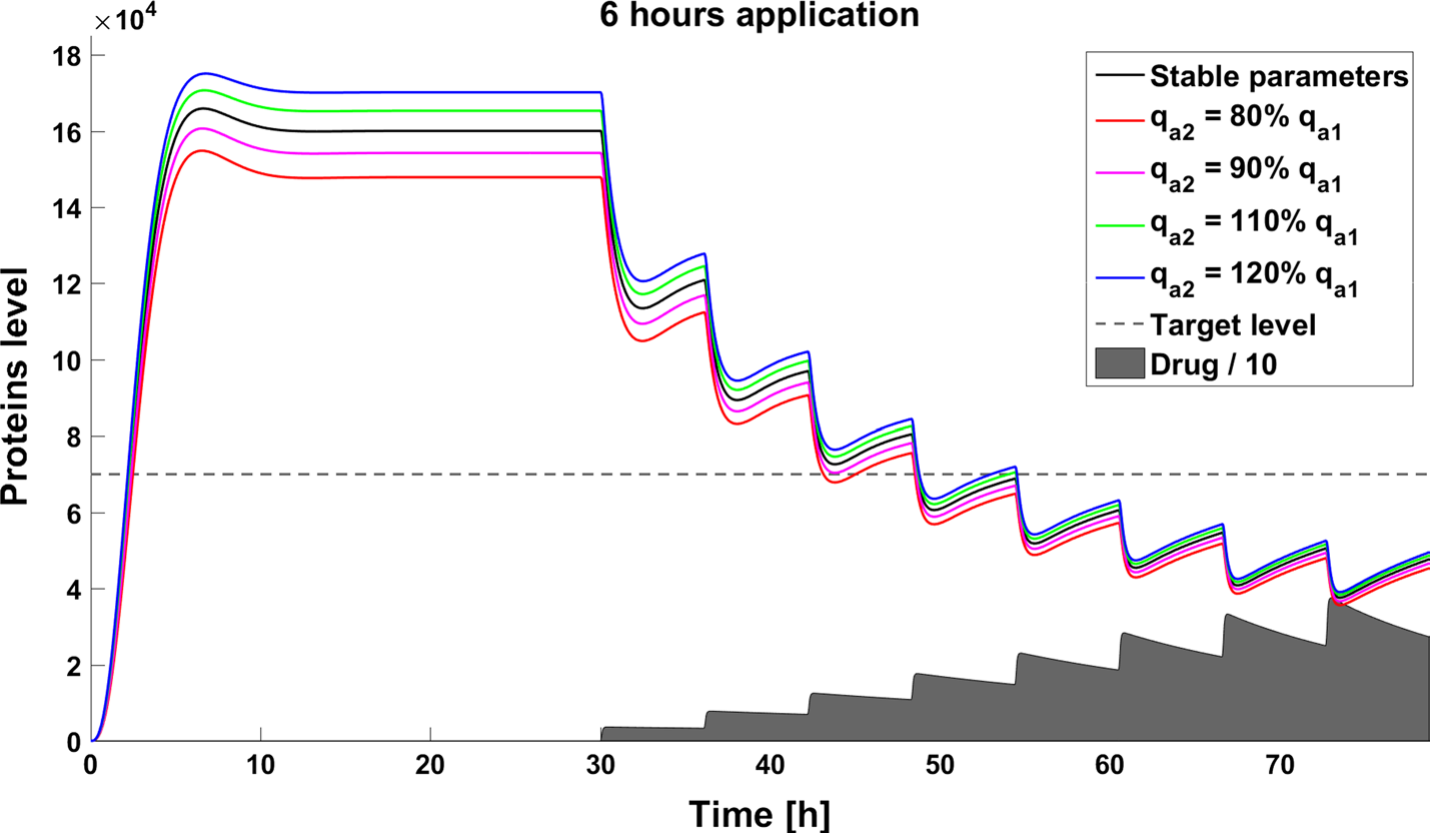
Time course of the level of target protein molecules in the model with a negative feedback loop with drug degradation after application every 6 h

**Fig. 18:**
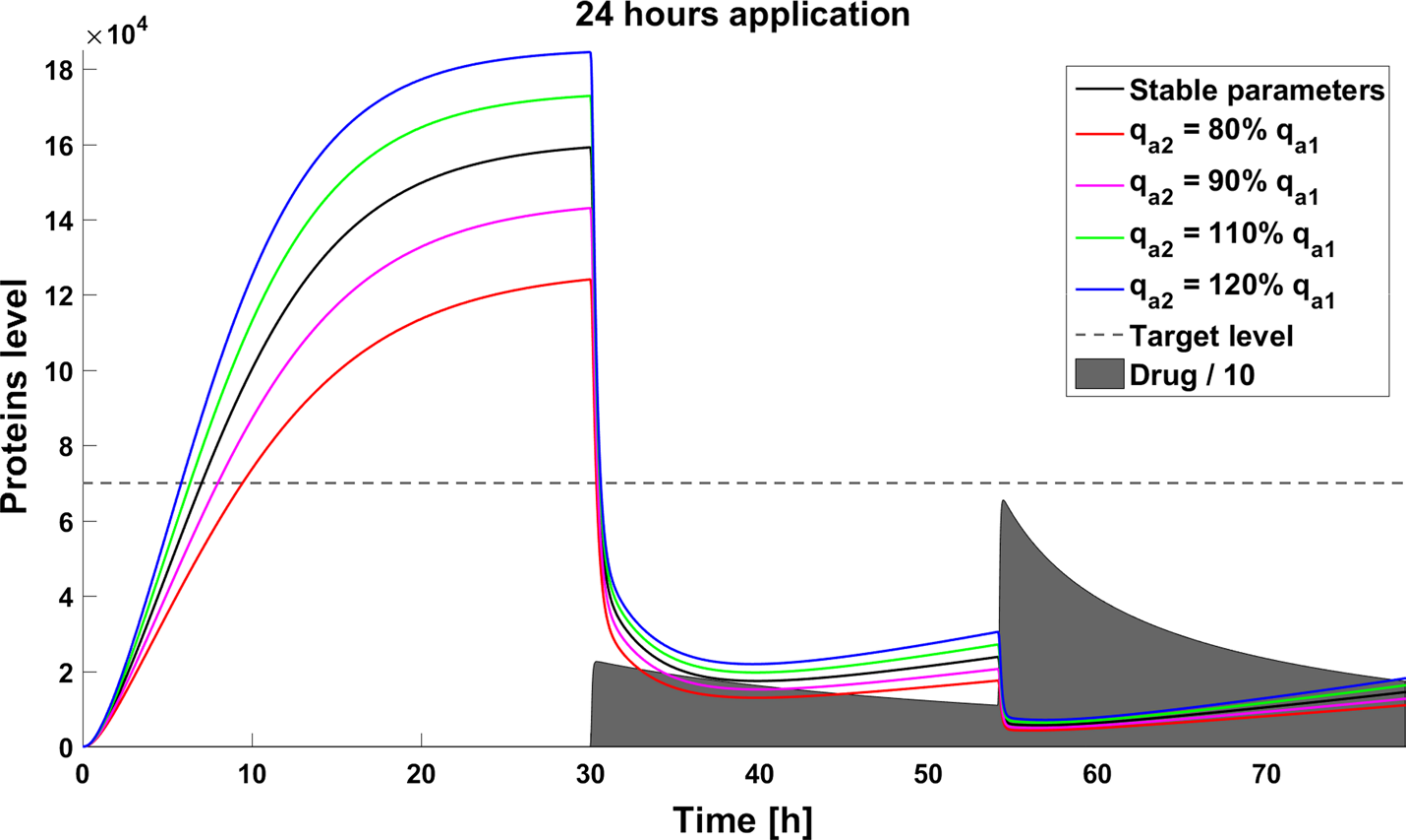
Time course of the level of target protein molecules in the model with a positive feedback loop with drug degradation after application every 24 h

**Fig. 19:**
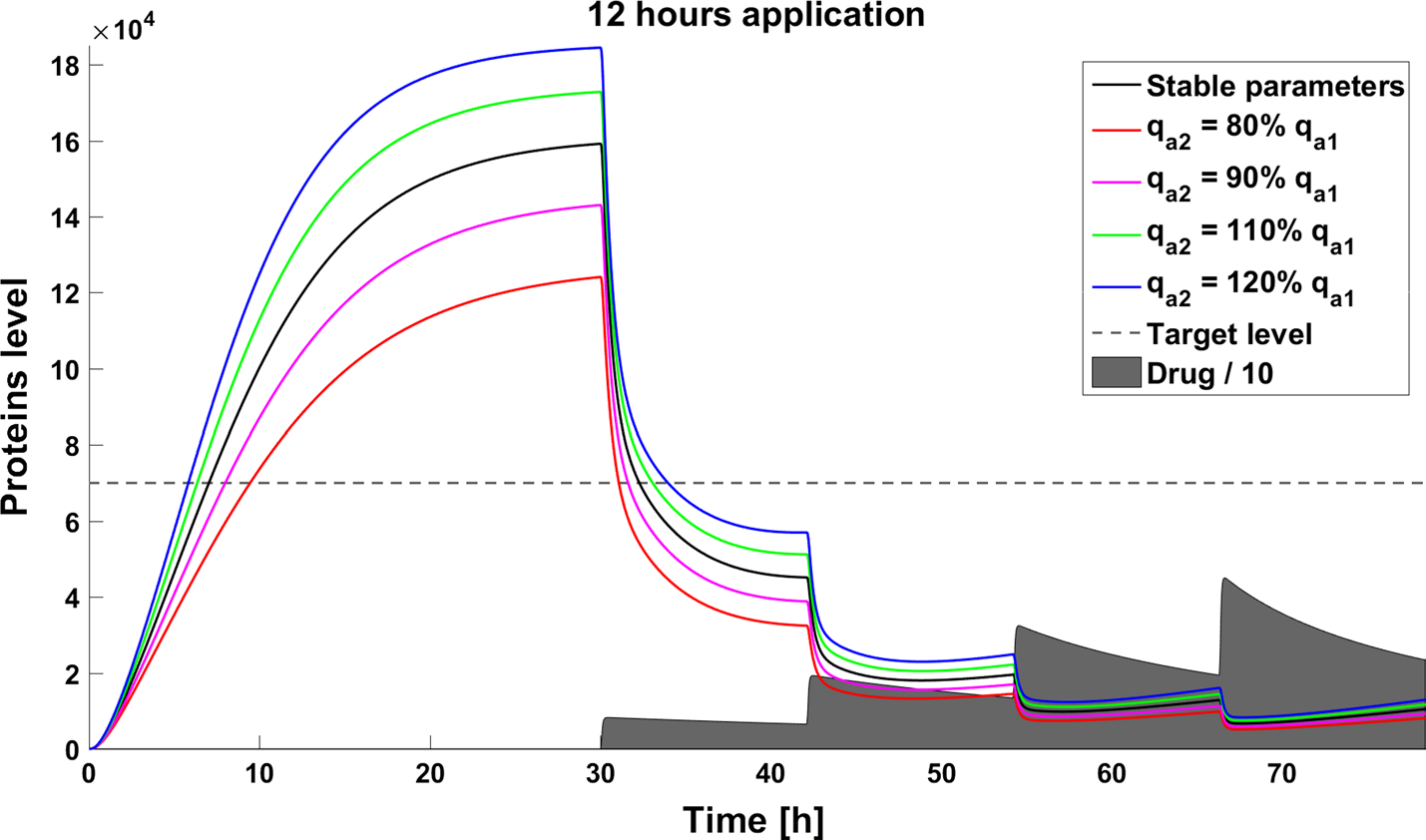
Time course of the level of target protein molecules in the model with a positive feedback loop with drug degradation after application every 12 h

**Fig. 20:**
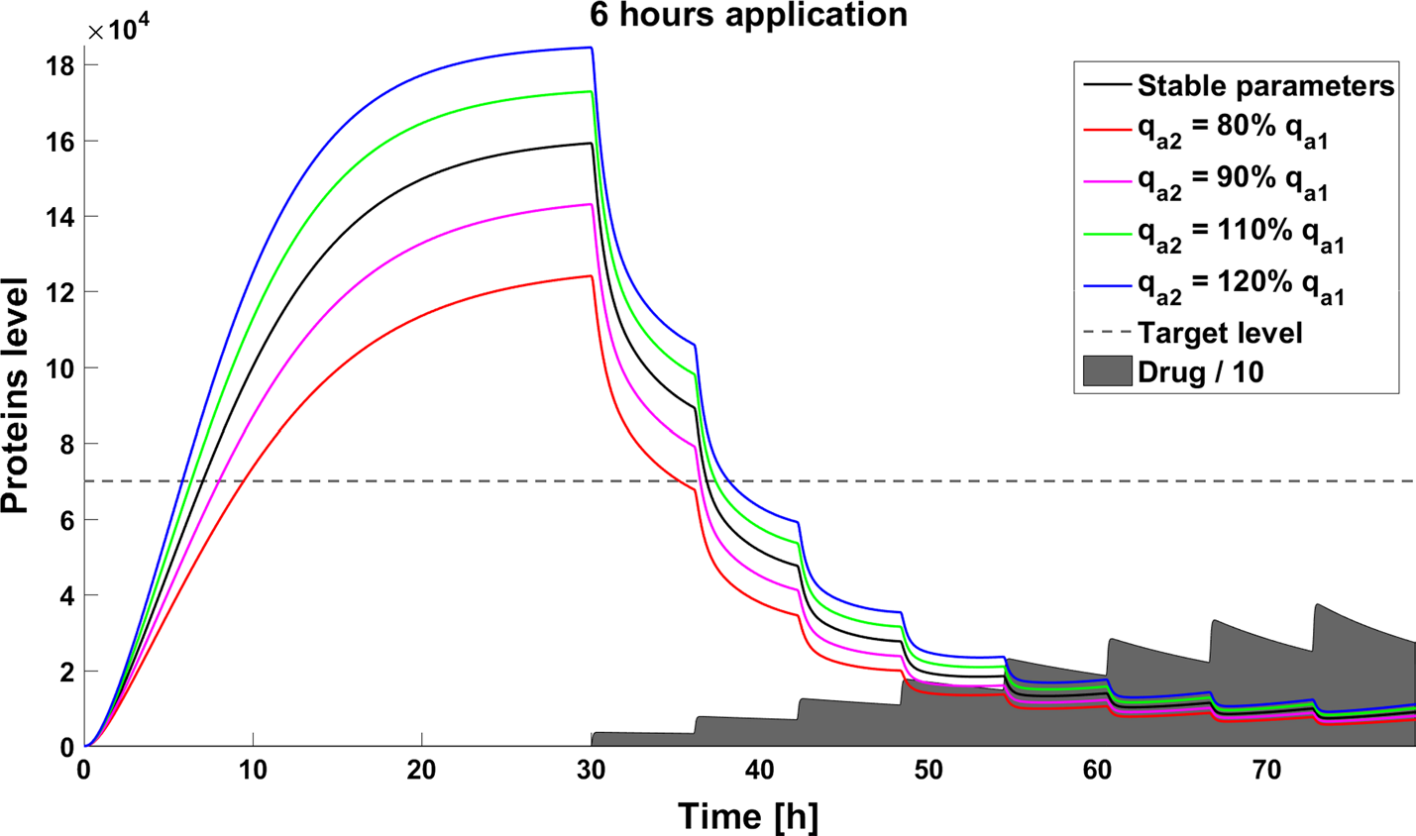
Time course of the level of target protein molecules in the model with a positive feedback loop with drug degradation after application every 6 h

When we take into account drug removal, its influence on the level of the target protein decreases with time. As a result, in all the models the protein level goes over the given threshold and therapy fails after some time (Fig. 11). To ensure successful therapy, a drug should be applied repeatedly and we consider next therapy with drug administration at different frequencies. We compare system results for the same drug dose, because the nonlinear process of drug removal influences the results in the case of different doses. In reference to real drug application system, we apply the same cumulative drug dose in all therapies. In the case of more frequent drug application we use accordingly smaller drug doses (*U*(*t*)): for application every 6 h the dose is 70, for every 12 h it is 140, and for every 24 h it is 280 arbitrary units. In the model without feedback loops, the time to reach the goal depends on the frequency of drug application. The high drug dose for application every 24 h is effective right after the first application, so the therapeutic goal is reached very fast (Fig. 12). In schedules with a smaller drug dose, a longer time is needed to decrease the target protein level under the threshold (Figs. 13, 14). In the model with a negative feedback loop the first application of a drug dose of 280 is not sufficient, and the target protein level exceeds the desired threshold quite rapidly; the second application after 24 h is needed for efficient therapy (Fig. 15). For this system, more frequent drug application will be preferable(see Figs. 16 and 17) because this allows the therapeutic target to be reached after about 19 h in the case of application every 6 h (Fig. 17). Like the experiments without drug degradation, the model with a positive feedback loop is the most sensitive for drug activity . The same drug dose induces a lower target protein level (Figs. 18, 19, 20) than those in the previous models. In the model with positive feedback, application every 24 h ensures that the goal is reached very rapidly.

The minimal therapy involves an application of the smallest effective drug dose to ensure the least toxic effect for the patient. Inaccuracy in determination of the model parameters can result in ineffective therapy, in this case due to an increased rate of gene activation. In the schedule with repeated drug application we use the normalized mean square error to calculate the discrepancy between the required and the observed protein level. The formula for this error is given by:

10 $$\begin{aligned} E = \frac{\sqrt{\sum {(A/A-\hat{A}/A)^2}}}{N}, \end{aligned}$$

where *A*—assumed protein level, $\hat{A}$—observed protein level and *N*—number of time samples.

**Fig. 21:**
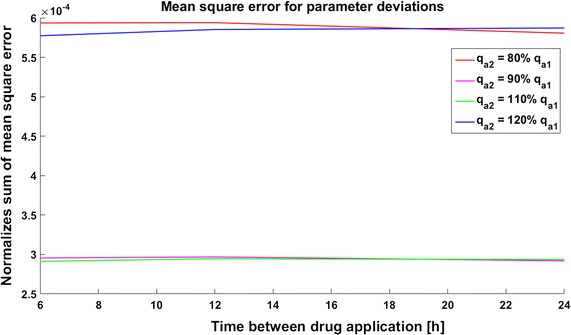
Time course of the level of target protein molecules in the model with a positive feedback loop with drug degradation. Mean square error for parameter deviations in different types of therapy

**Fig. 22:**
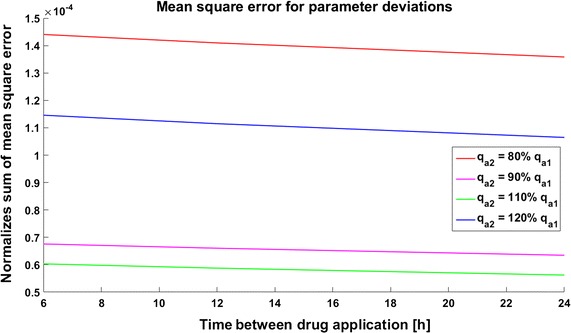
Time course of the level of target protein molecules in the model with a negative feedback loop with drug degradation. Mean square error for parameter deviations in different types of therapy

**Fig. 23:**
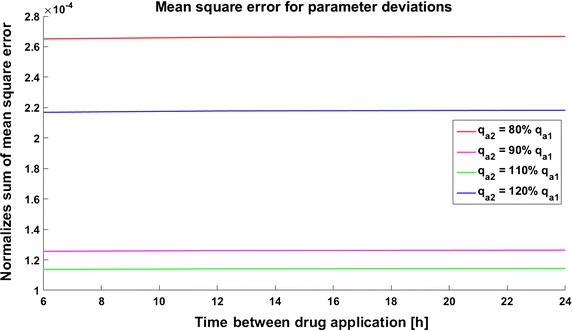
Time course of the level of target protein molecules in the model without a feedback loop with drug degradation. Mean square error for parameter deviations in different types of therapy

The discrepancies are largest in the model with a positive feedback loop, where the mean square error is approximately $6*10^{-4}$ for 80 and 120% parameter perturbations (Fig. 21). In the model with negative feedback the mean square error is the smallest, and even in the case of 80% perturbation it is smaller than $1.5*10^{-4}$ (Fig. 22). In the model with a positive feedback loop the mean square errors for 80% and 120% perturbation are almost the same, but in the two other models the 120% perturbation results in a smaller error than the 80% perturbation (compare Fig. 21 with Figs. 22, 23). In the system without feedback and in that system with positive feedback discrepancy does not depend on the frequency of drug application. In the model with negative feedback, less frequent drug administration results in a smaller discrepancy.

## Discussion

Systems with switching form a quite common and well investigated group of automatic control problems [27, 28]. However, application of methods known from control theory to real biological systems with stepwise changes of parameter values is quite a new approach. In the present work abrupt changes of parameter values for a target protein are used to model the results of application of a therapeutic drug. In the case of the drug degradation multiple drug dosage case can be treated as a multiple switch in the system. This property creates a variety of possible applications, for example cell disorders like carcinogenesis which induce many different changes in cellular processes can be modeled as switches in the structure of the system. All the models presented in this work, even those with feedback, are very simplified, and we do not consider any specific target protein. In biological systems, dependencies between proteins and other molecules create very complex regulatory networks which can include multiple different feedbacks, both positive and negative. Moreover, in more complicated systems we can observe delays in signal transduction so that the dynamics can be even more complicated. It is even possible that complicated dynamics of a real system will filter out the effect of parameter perturbations so that no effect will not be observed at all. Analysis of real systems can therefore be challenging, but in our opinion it is worth performing. However, in this work we neglect the complicated real-life intercellular dynamics to focus on the problem of the effects of parameter perturbations in the system. Efficient therapy usually requires the application of several drugs. The pharmacodynamics and pharmacokinetics of particular drugs, especially their removal from and inactivation by the organism, enforce frequent drug application to maintain the desired effect and due to drug toxicity and side effects, the cumulative dose should be minimized. In the case of similar target protein levels in the steady state, the internal feedback can result in decreased or increased sensitivity to the drug (Fig. 2). The effects observed here emphasize the importance of careful investigations of the regulatory networks inside the cell. An optimal algorithm for drug application is still a problem which has not been fully examined. High drug doses can be toxic for patients, and moreover they are very quickly removed from the body due to the nonlinear process of drug excretion. Side effects can be minimized by application of smaller drug doses with higher frequency, but the dynamics of the process is very important. The time to reach the therapeutic goal can be a crucial factor in selection of a therapy and in the case of fast and insensitive systems such as those with negative feedback studied here, more frequent drug application allows the therapeutic target to be reached much earlier than with a schedule of less frequent applications with the same cumulative dose. Inaccuracies in parameter determination and times of drug administration could have a significant impact on the results of therapy, and thus should be always taken into consideration. Attempts have been made to estimate the influence of parameter deviations on the reachability of the therapeutic goal using the analytical approach [29]. Parameter perturbation will be especially crucial for systems where switching depends on the system’s state. If the switches are used for modeling the internal features, for example step-like activation of a protein by other macromolecules, perturbations in the model parameters will influence not only the numbers of molecules but also the time of the switch, and as a result such models will have significantly different dynamics. In such case the problem of parameter perturbation will be even more interesting and thus worth focussing on. In this paper, despite the existence of genes in our models we have focused only on the deterministic approach. The reason was that the majority of published models is still deterministic, and we want to point out some possible problems related to the determination of parameter values (and thus their fluctuations) when systems with switchings (such as drug application) are considered. This leads us to results which may be considered as the expected values realized in a real stochastic system. One should remember, however, that the real response of a single cell to a drug may be more strongly affected by the intrinsic noise resulting from stochastic gene switching than by parameter deviations, and this possibility will be the subject of our future studies. The other interesting phenomena related to stochastic gene switching and drug efficiency was pointed out in our previous work [24] where we show that the drug dose required to achieve the therapeutic goal may be significantly higher when a system is modeled by the stochastic approach than by the deterministic approach. In real biological systems, differences in parameter values can be mitigated by the very complex dynamics of non-linear systems and the stochasticity of processes such as gene activation. Nevertheless, numerous models presented in the literature were created as deterministic systems, so the problem discussed in this paper is applicable to them.

## Conclusions

Creation of models of biological systems can be very useful and thus becomes a more and more popular method for examination of biological processes. One of the biggest problems in model development is determination of the parameter values. Some parameter values are calculated based on biological experiments, which are often very expensive, time consuming and gives only approximate results. However, the values of many biological parameters can not be calculated directly and therefore must be estimated. Moreover, the rates of biological processes are dynamically changing due to the physical, chemical and biological conditions inside the cell or organism. Taking all these factors into account, the problem of perturbations in model parameters is a significant issue. In this work we focus on the influence of parameter deviations on the reachability of a therapeutic goal in quite simple models of a therapeutic drug targeting a specific protein in vivo, and we examine the general properties of these models. Negative feedback in the model decreases the sensitivity to a switch (in this case the application of the drug) but also decreases the sensitivity to parameter perturbations. This model has very rapid dynamics and its response shows overregulation. On the other hand, a model with positive feedback is characterized by very slow changes. It is very sensitive to the schedule of drug application, but perturbations in the model parameters result in significant difference in the output and, moreover, changes in the parameter values can make the model unstable. Considering changes in the level of active drug due to its degradation, multiple drug application is optimal. More frequent drug application allows for using a smaller total dose, so that the therapy can be effective and has lower toxicity. A high drug dose results in a very large and rapid jump of drug concentration which could cause serious damage to the whole body. The frequency of drug application has a significant influence on the time to reach the therapeutic target. In our opinion, analysis of dynamics and sensitivity for the models of such processes plays a crucial role in their application
